# Scaling up mental health services in Sudan: Sudanese psychiatrists’ opinions

**DOI:** 10.1192/bji.2020.17

**Published:** 2020-11

**Authors:** Abdelgadir Hussein M. Osman, Aisha Bakhiet, Samia Elmusharaf, Abdelaziz Omer, Abdalla Abdelrahman

**Affiliations:** Department of Psychiatry, Faculty of Medicine, University of Khartoum, Khartoum, Sudan. Email: abdelgadir1159@yahoo.com

**Keywords:** Scaling up mental health services, capacity building, mental health, Sudan

## Abstract

We invited 108 psychiatrists of Sudanese origin, working in and outside Sudan, to take part in a study looking at the most appropriate method for scaling up mental health services in Sudan. Of those psychiatrists who were approached, 81 (75%) responded. Among the respondents, 30 (37%) resided and worked in Sudan, and 51 (63%) worked outside Sudan (mostly in the UK and Arab Gulf States). Most respondents preferred the lay counsellor model (43, 53.2%) to address the current shortage of human resources for scaling up mental health services.

## Background

Several World Health Organization (WHO) reports have issued calls for low- and middle-income countries to scale up the mental health components of their healthcare systems, particularly the skilled workforce.^1–4^ Sudan's mental health policy, last formulated in 2008, showed that there was a huge gap in the mental health workforce. It estimated that the total number of individuals working in mental healthcare facilities (including those in private practice) was 0.92 per 100 000 population. The breakdown according to profession was as follows: 0.06 psychiatrists, 0.09 other medical doctors, 0.12 nurses, 0.13 psychologists, 0.08 social workers and 0.45 other health workers per 100 000 population.^4–6^ There is an uneven distribution of human resources in favour of a few big cities and the capital city of Khartoum. Only seven out of 18 states in Sudan have psychiatric treatment facilities (hospitals or in-patient units).

Researchers and policy makers emphasise the need for low-income countries to scale up their human resources to provide a minimum of 22.3 mental health workers per 100 000 population.^1–3^ Consequently, the projected mental health human resources in Sudan would need to be increased to 8474 (22.3 × 10 × 38) mental health workers, for a population of 38 million. Sudan's current workforce is estimated to be 530, working in both the public and private sectors. To bridge this gap, Sudan needs to increase the number of currently practising mental health workers by 16-fold in order to fulfil the minimum required workforce to provide good mental health services. The WHO acknowledges that in low-income countries the ratio of people who need mental healthcare to the number of qualified psychiatrists is so disproportionate that there is no prospect of an adequate number of psychiatrists being able to deliver the care that is needed in the foreseeable future.^3–5^ In Sudan, this lack of support has resulted in a high prevalence of mental disorders among its 38 million citizens, compounded by an impoverished economic situation resulting in a scarcity of essential psychotropic drugs. Thus, there are huge challenges for mental health leaders in Sudan to scale up existing human resources to provide capacity in mental healthcare.^4–6^

## Method

This project came about following a series of workshops, brainstorming meetings and conferences attended by government officials with ministerial capacity, policy makers, and senior psychiatrists working inside and outside Sudan (e.g. the UK and Arab Gulf States). The first workshop was held in January 2015 and the last was held in September 2016. The main themes of all the workshops were about capacity-building. There was discussion about which service models would be most appropriate to provide resources for patients and their families, especially the less advantaged population and those living in remote areas. This discussion resulted in ideas-gathering (modified Delphi approach) and highlighted methods for scaling up services.^3^ No formal research has yet been published in this area to inform the country's future direction.

### Study design

This study was a cross-sectional survey of psychiatrists of Sudanese origin currently practising in or outside Sudan. Mixed qualitative and quantitative methods were used for data collection.

### Instrument and measurements used

A simple questionnaire was designed to gather personal characteristics about the participants, including age, current place of employment (i.e. in or outside Sudan) and reasons for current place of employment. The second section of the questionnaire was a modified Likert-type rating scale that used fixed choices of responses, designed to measure participants’ levels of agreement or disagreement with models of capacity building. They were given four non-exclusive choices for capacity building (training more medical doctors, nurses, psychologists and social workers, or lay counsellors) to fill the gap in mental health services’ needs. Participants were also given a fifth optional section for stating further remarks and suggestions.

### Sample selection technique

Data concerning registered psychiatrists in Sudan were obtained from the Sudan Medical Council and the Sudan Psychiatrists Association for current active and working psychiatrists in Sudan. For expatriate psychiatrists there were no comprehensive formal lists; therefore, we approached all known bodies representing expatriate psychiatrists of Sudanese origin. These included the Sudanese Psychiatric Association, based in the UK, together with the professional and social network of Sudanese psychiatrists practicing in the Arab Gulf States, in order to reach individuals working in Arab countries. Furthermore, all participants were asked to nominate untapped psychiatrists in other countries.

The research questionnaire and separate background information notes were emailed to all database psychiatrists of Sudanese origin. Two more reminders were sent later to non-responders. A special blog on social media (WhatsApp) was designed for interaction and information exchange.

### Data analysis

Data were analysed using SPSS version 22.0 to generate quantitative statistical measurements. Results are expressed as frequency tables, basic comparison tables and percentages. The chi-square test was used to determine significance and *P*-values.

### Consent and ethical approval

This study was reviewed and approved by the ethics committee of Khartoum Medical School. As this research was conducted with the full participation of senior psychiatrists, the ethics committee waived the need for written consent and considered participation to imply consent by default.

## Results

Of a total of 108 psychiatrists of Sudanese origin who were approached to take part in the study, 81 (75%) responded by completing the required questionnaire. There were no comprehensive lists or sources for psychiatrists of Sudanese origin outside Sudan, but it is believed that the number is far higher than this. Forty-five of the respondents (55.6%) were male and 36 (44.4%) were female. Most of the participants (44, 54.3%) were aged 36–50 years, 13 (16%) were ≤35 years, 23 (28.4%) were 51–65 years and one was ≥66 years.

A total of 30 (37.0%) participants resided and worked in Sudan, 26 (32.1%) worked in the Arab Gulf States, 24 (29.6%) worked mainly in the UK and other European countries, and one worked in none of the stated countries. The most common reason for relocation was financial (37%), and roughly equal proportions (27 and 24%, respectively) relocated for family or professional reasons.

Table 1 shows the preferred method for scaling up mental health services and filling the mental health worker gap in Sudan so that most regions and districts would have the appropriate mental health personnel.

**Table 1:** Participants’ views on preferred method of scaling up mental health services in Sudan

Preferred training method	Frequency	%	*P*-value
Medical doctors			
Strongly disagree	39	48.1	0.000
Mildly disagree	6	7.4	
Mildly agree	1	1.2	
Strongly agree	30	37.0	
Nurses			
Strongly disagree	48	59.3	0.000
Mildly disagree	1	1.2	
Mildly agree	1	1.2	
Strongly agree	30	37.5	
Psychologists/social workers			0.000
Strongly disagree	42	51.9	
Mildly disagree	03	3.7	
Mildly agree	01	1.2	
Strongly agree	31	38.3	
Lay counsellors			0.001
Strongly disagree	35	43.2	
Mildly disagree	01	1.2	
Mildly agree	01	1.2	
Strongly agree	43	53.1	

## Discussion

This study showed a clear trend, which was consistent with other evidence of a ‘brain drain’ of psychiatrists from low-income countries. Not only do the majority of Sudanese psychiatrists emigrate abroad but, more alarmingly, this trend is unlikely to be resolved in the foreseeable future, as indicated by the outcome of this study and another recently published article in this journal on Sudan's mental health profile.^4^ It is not surprising, therefore, that most participants in this study favoured training lay counsellors in order to expand and scale up mental health services in Sudan rather than training more doctors. The latter are unlikely to settle in the country, let alone cover the mental health needs of our population in remote geographical areas. This lay counsellor model would be expected to function as part of a multidisciplinary team using the stepped care model of support. Similar models have been tried elsewhere, in Nigeria, Chile, Ethiopia and Uganda.^7,8^ Task shifting of responsibility from psychiatrists to community health workers and community lay counsellors has also been reported to be effective in increasing the mental health workforce in low-income countries.^6–8^ This model was advocated by most of the psychiatrists we surveyed (43, 53.1%) and is seen as strategically essential to expand and scale up Sudan's mental health services. Moreover, this model could broaden service coverage to areas that otherwise would not see any workforce input in the near future. Although it would lead to a relative decline in the proportion of highly skilled staff, this could be ameliorated through additional training. Lay counsellors could increase the skill mix of the mental health workforce at a community level and thereby expand the mental health coverage for the population. This model of care is an effective method for reaching remote areas that otherwise would not have any mental health input.

The challenge of scaling up mental health services in Sudan is deciding what to implement rather than how to implement change. There is already robust evidence for a range of cost-effective interventions but little evidence about how these might be delivered in diverse low-resource settings. We believe the lay counsellor model would be financially affordable and provide timely use of services according to need in a geographically huge country.^7–9^

Geographical access is an important part of any effective model of healthcare. Geographically remote areas are most severely disadvantaged in terms of service availability, and this represents an important barrier to psychiatric service use.^7,8^ Accordingly, any expected model of service expansion that aims to reduce barriers to service availability should be practical, affordable, sustainable and efficacious (i.e. it should achieve what it is designed for). A similar model has been tried in Nigeria, Ethiopia and Chile, although in the latter case it was more specifically focused on depression diagnosis and treatment.^8–10^

Many challenges are expected with the lay counsellor model. There will be a need for intensive training to provide proper recognition and identification of mental health problems, especially in psychotic and complicated cases. On the other hand, once lay counsellors have been trained, they could be placed strategically in mental health disorder hot spots, such as camps for internally displaced persons, geographically remote areas and religious camps (khalwas).^10–12^

## Conclusion

The majority of Sudanese psychiatrists that took part in this study advocated scaling up mental health service in Sudan via a lay counsellor model, which is believed to be suitable for a large under-resourced country and could provide a sustainable, affordable service. Most senior psychiatrists of Sudanese origin emigrate and live abroad for financial, personal and professional reasons. We therefore recommend that the Department of Health in Sudan is urged to galvanise efforts to create a collaborative, integrated mental healthcare system, to improve national coverage and to overcome the deficiencies that currently exist.

## Data Availability

Data can be requested from the corresponding author.

## Supplementary material

For supplementary material accompanying this paper visit https://doi.org/10.1192/bji.2020.17. click here to view supplementary material
